# Warming effects on the urban hydrology in cold climate regions

**DOI:** 10.1038/s41598-017-05733-y

**Published:** 2017-07-19

**Authors:** L. Järvi, C. S. B. Grimmond, J. P. McFadden, A. Christen, I. B. Strachan, M. Taka, L. Warsta, M. Heimann

**Affiliations:** 1 Department of Physics, University of Helsinki, Helsinki, Finland; 2 Department of Meteorology, University of Reading, Reading, United Kingdom; 3 Department of Geography, University of California, Santa Barbara, USA; 4 Department of Geography/Atmospheric Science Program, the University of British Columbia, Vancouver, Canada; 5 Department of Natural Resource Sciences, McGill University, Montreal, Canada; 6 Department of Geosciences and Geography, University of Helsinki, Helsinki, Finland; 7 Department of Built Environment, Aalto University, Espoo, Finland; 8 Max-Planck Institute for Biochemistry, Jena, Germany

## Abstract

While approximately 338 million people in the Northern hemisphere live in regions that are regularly snow covered in winter, there is little hydro-climatologic knowledge in the cities impacted by snow. Using observations and modelling we have evaluated the energy and water exchanges of four cities that are exposed to wintertime snow. We show that the presence of snow critically changes the impact that city design has on the local-scale hydrology and climate. After snow melt, the cities return to being strongly controlled by the proportion of built and vegetated surfaces. However in winter, the presence of snow masks the influence of the built and vegetated fractions. We show how inter-year variability of wintertime temperature can modify this effect of snow. With increasing temperatures, these cities could be pushed towards very different partitioning between runoff and evapotranspiration. We derive the dependency of wintertime runoff on this warming effect in combination with the effect of urban densification.

## Introduction

The most widely studied urbanisation-induced environmental impacts are the urban heat island effect^1, 2^ and increased flood risk^3^. Such studies have mainly focused on the mid-latitudes and tropics, whereas cold climate cities remain virtually unstudied despite the fact that cold regions are predicted to be highly sensitive to climate change^4^. In the Northern hemisphere, snow covers wide land areas (on average 588 400 km^2^ in 2007–2010, Supplementary Fig. S1), but significant decreases in high latitude snow cover duration and spatial extent have been observed over recent decades^5–7^. This has induced peak flows and more runoff throughout winter in snow-melt hydrology dominated regions^8–10^. Furthermore, climate projections suggest that a reduction in snow cover will continue in the future^11^. Although global warming has regional scale consequences for air temperatures and ecosystems^12, 13^, one of the greatest gaps in current knowledge about the interaction between global and urban climatology in these regions is how changes in snow cover will impact the urban environment at the local (within city) scale. In addition to buffering wintertime surface runoff^14–17^, snow modifies urban surface properties via changes in albedo, thermal insulation and water availability, thus its contributions to the urban hydrological cycle are of principal importance. Furthermore, changes in the hydrological cycle will impose requirements for effective stormwater systems that are adaptive and resilient to sudden and extreme climatic changes, and affect the mobility of pollutants and in-stream chemistry^18^ and their seasonal distribution^19^ modifying the environmental burden on aquatic ecosystems^20, 21^.

In cold climate cities the potential damage caused by surface flooding to existing infrastructure, creating large economic impacts, is of great concern^22, 23^. In the European Union alone, the estimated annual flood loss was 4.9 billion euros in 2000–2012^24^. Extreme precipitation events in a warming climate^25–27^ together with land cover changes^17^ are generally considered to be the causes of increased flood risk in urban areas. Since the greatest climate changes are expected in wintertime in northern regions, the effect of climate variability will be especially important for cold climate cities.

**Figure 1:**
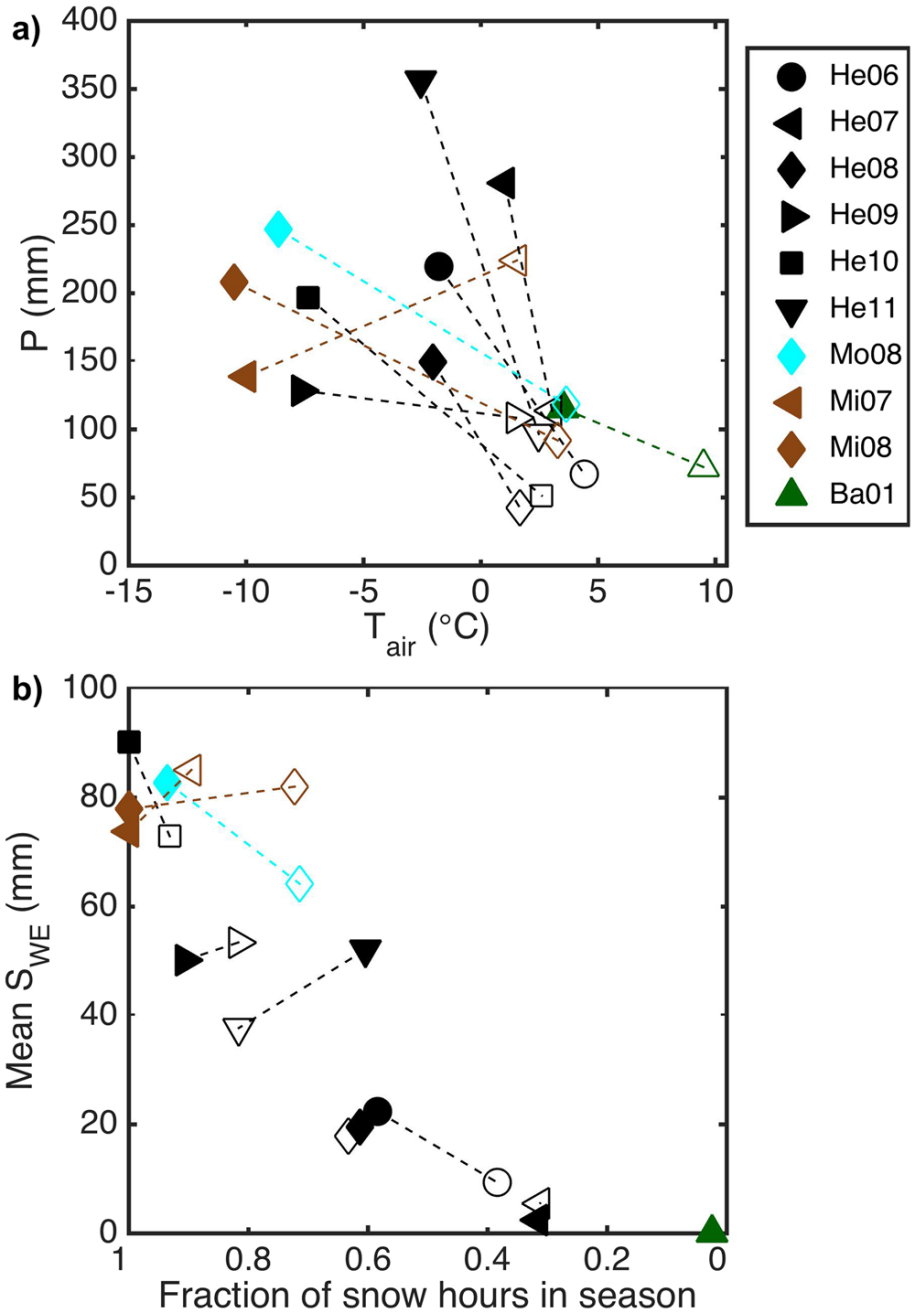
Winter and spring time climatology of the studied areas. (**a**) Observed mean air temperature (*T*_*air*_) and total precipitation (*P*, including both liquid water and snow) and (**b**) modelled mean snow water equivalent (*S*_*WE*_) and fraction of snow hours for winter months (December–February, solid symbols) and spring months (March–May, open symbols). To aid interpretation, dashed lines connect the seasons for each site (He – Helsinki, Mo- Montreal, Mi- Minneapolis, Ba-Basel) and year (2 digits). For data sources see Supplementary Table S1.

Here we propose a framework to assess the combined impact of climate and urbanisation on the urban hydrological cycle across the distinct seasons in cold climate cities. We use an urban hydrological model SUEWS^28–30^ (the Surface Urban Energy and Water balance Scheme) in combination with energy and water exchange observations to evaluate these dependencies at eleven study areas in four cold region cities in Europe and North America (Helsinki, Finland; Basel, Switzerland; Montreal, Canada; and Minneapolis-Saint Paul, USA). The dependence of surface runoff and evapotranspiration on urbanisation and climate in these cities is analysed over multiple years with varying climatology (Fig. 1) and surface cover characteristics (Supplementary Table S1).

### Response of hydrological components to impervious cover

**Figure 2:**
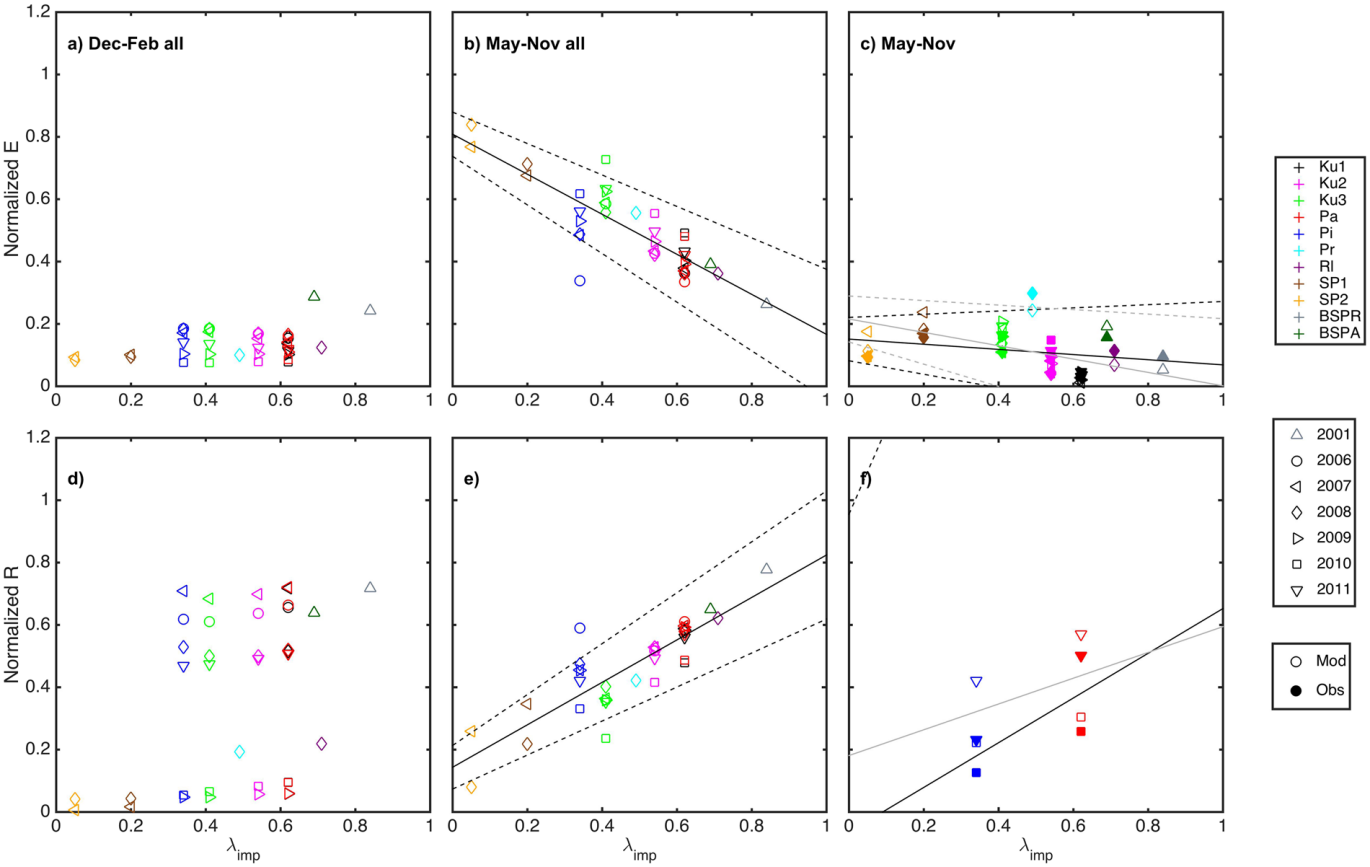
Hydrological components with impervious cover. Cumulative (**a–c**) evapotranspiration (*E*) and (**d–f**) surface runoff (*R*) as normalised by cumulative precipitation and irrigation. (**a,d**) show modelled (open symbols) winter (December–February) and (**b,e**) snow-free (May–November) months for all hours and sites (colours) on different years (symbols), and (**c,f**) modelled (open symbols) and observed (solid symbols) snow-free (May–November) months for sites with observations (in cumulative normalised *﻿﻿﻿E* and *R* only hours with observed data are used). Linear regression fitted to the modelled (black) and observed (grey) data are shown with the 95^th^ confidence limits (dashed lines). See Supplementary Table S2 for fitted coefficients.

We first quantified the strong response of evapotranspiration (*E*) and surface runoff (*R*) in snow-free months (May–November), each normalised by total precipitation and irrigation within the period, to the impervious surface cover (λ_imp_) (Fig. 2b,e). The responses are based on modelled data, as cumulative values are not possible to be calculated from the observations because of large amounts of missing data. The studied sites range from a golf course in Minneapolis (SP2) as the least urbanised site (5% impervious surfaces) to Basel (BSPR) (84% impervious surfaces). These first-order relations indicate that a 20% increase in the impervious surface cover fraction with the same precipitation would result in an 8% increase in seasonal cumulative runoff and a 50% increase would lead to a 17% increase in cumulative seasonal normalised runoff. These responses are confirmed by observations (Fig. 2c,f). In winter months (December–February), the inter-annual variability of the weather dominates the behaviours of both *E* and *R* (Fig. 2a,d) and the largest normalised cumulative runoffs (0.68–0.72) are seen in the warmest winters (Helsinki 2007 and Basel 2001) and the smallest (less than 0.06) in the coldest winters (Minneapolis 2008 and 2009, and Helsinki 2009 and 2010). In March–April the hydrological cycle is dominated by weather and melting of snow rather than the impervious surface cover fraction (Supplementary Fig. S2). This shift from impervious surface fraction to weather-dependence is mainly due to the presence or absence of snow.

### Response of hydrological components to climate and impervious cover

**Figure 3:**
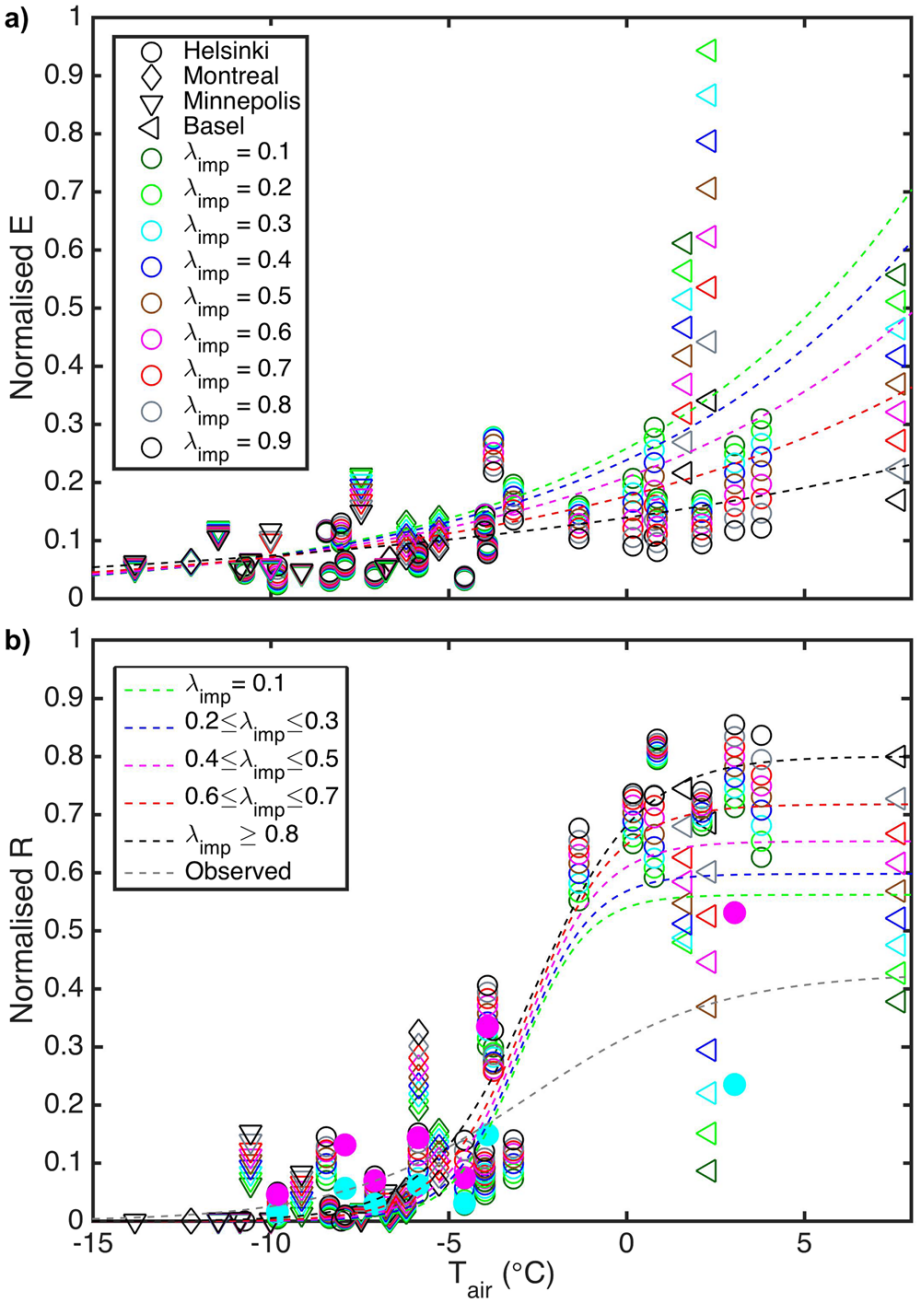
Wintertime monthly hydrological components. (**a**) Modelled monthly evapotranspiration (*E*) and (**b**) modelled (open symbols) and observed (solid symbols) monthly surface runoff (*R*) normalised by monthly precipitation plus initial snow amount as a function of air temperature (*T*_*air*_) and impervious surface fraction (λ_imp_) in December–February. The impervious surface cover  in each city was increased with 10% bins between 10% and 90%. The fitted curve has an exponential form (${aexp}(b{T}_{air})$) for the normalised evapotranspiration, and a logistic form ($\frac{a}{1+\exp [-b({T}_{air}-c)]}$) for the normalised runoff (see Supplementary Table S3 for coefficients).

We further explored the dominant factors of the hydrological cycle during the winter months (Dec–Feb) using sensitivity runs where the amount of impervious surfaces in each city was varied (Fig. 3). The winter monthly mean air temperature largely explains the magnitude of normalised runoff at the studied years with warmer months generating more surface runoff (Fig. 3b). This is further enhanced with increased impervious surface cover as snow clearing and transport decreases the amount and duration of snow on the ground resulting in patchier snow cover. The decrease in snow water equivalent due to snow clearing varies from 82 mm (90% impervious cover) to near zero (10% impervious cover) with greater reduction at sites with most snow with impervious fraction of 10%. The maximum reduction in the duration of the snow covered period due to snow clearing is six days as seen in Helsinki 2011.

Runoff enhancement is pronounced at lower air temperatures when more snow in the study region is common, and less at higher air temperatures when the amount of impervious cover starts to become dominant. Surface runoff is initiated in winter if the mean monthly air temperatures are ≥ −10 °C. When mean monthly air temperatures exceed this threshold, the typical temperature distribution has sufficient number of warm days such that liquid precipitation events and melt of snow cover begin to produce runoff. At −5 °C, 8% of the cumulative precipitation will be directed to runoff when the impervious fraction is 40–50% or, with the same precipitation but 80–90% impervious cover, 13% of water will leave as runoff. Similar relations are found from analysis of all winter months and using two-week periods (Supplementary Table S3). Clearly, a greater fraction of water goes to *R* than to *E* during the winter months. Higher air temperatures have a smaller effect on *E* than *R*, because the former is rather limited by the amount of active vegetation, which is in its minimum over the winter months due to frozen ground, low solar radiation and leaf area index, than the available water. The small *E*, in combination with minimum water infiltration due to the frozen ground, increases the risk of surface flooding in winter conditions as more precipitation is directed to runoff.

### Increase in intense runoff events

**Figure 4:**
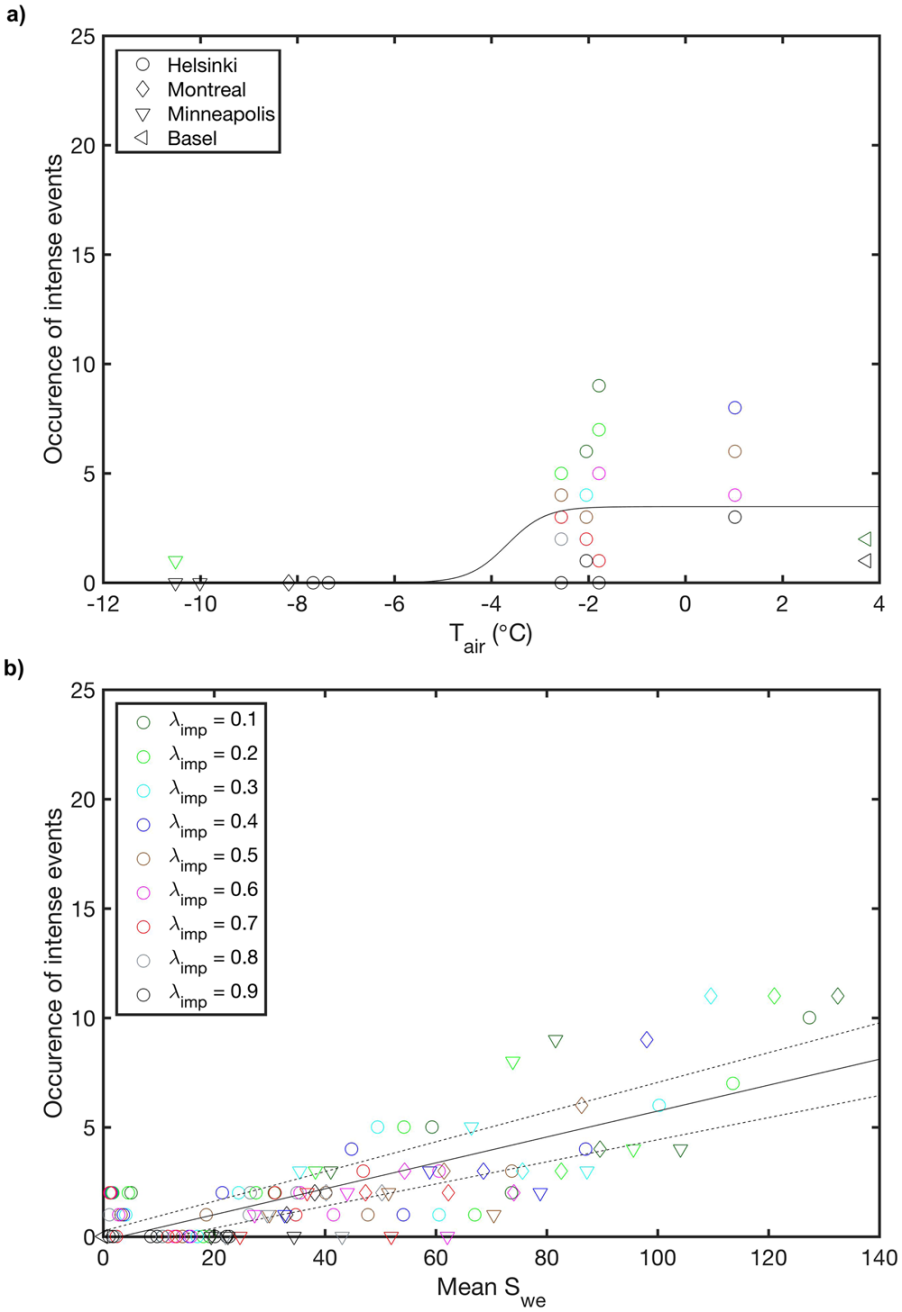
Occurrence of intense (>95^th^ percentile of all analysed sites and years) normalised daily runoff (*R*) events. The modelled occurrence of intense events (**a**) as a function of air temperature (*T*_*air*_) during winter (December–February) and (**b**) as a function of mean snow water equivalent (*S*_*WE*_) during the thermal melting period (mean monthly air temperature between 0–5 °C). The impervious surface cover  in each city was increased with 10% bins between 10% and 90%. Solid lines are the least square interpolations to the data points (logistic function and linear fit, respectively) and dashed lines represent 95% confidence. See Supplementary Table S3 for fitted coefficients.

In addition to the increase in cumulative normalised runoff, we show how the air temperature affects the number of intense normalised daily runoff events (i.e. normalised daily runoff >95^th^ percentile across all sites and years) over the winter months (Fig. 4a). The dependency follows a similar logistic function to the cumulative values, but the impact of increasing impervious cover is less pronounced (possibly caused by the smaller number of data points). Extent of impervious cover and snow amount both appear to impact the cumulative normalised runoff and the occurrence of intense normalised runoff events during the melting period (i.e. when mean daily air temperature is between 0–5 °C) (Fig. 4b). The normalised runoff is greater in urban areas with less impervious cover as there will be a larger snowpack retained to be melted. Thus the ratio of impervious to pervious area modifies the likely timing and amount of runoff as denser urbanisation modifies the snow amount and causes earlier (winter) runoff, and reduces the springtime daily runoff events.

## Discussion

Using a combination of modelling and observations, we show that in cold climate cities, both weather conditions and amount of impervious cover modify the urban hydrological cycle components that are key to urban planning and sustainable design. Without snow, the local hydrological exchanges are strongly controlled by the built-vegetation partitioning, whereas snow buffers the surface runoff and affects water availability for runoff. The analysis identifies a new key element to urban warming effects: increases in wintertime surface runoff in cold climate cities due to modifications in the spatial extent and duration of snow cover. This warming related modification of the wintertime water cycle, with increased water stress for urban environments and corresponding environmental burden, should be expected to accompany warming of high-latitude regions with climate change.

Our results show that it is critical to assess the climate sensitivity of the output generated by hydrological prediction tools such as SUEWS, to understand their robustness, so they can be implemented in sustainable urban design applications in a changing climate. Urbanisation does not automatically increase the surface runoff in cold climate cities, rather its magnitude and timing are complex functions of snow accumulation and clearing, the extent of snow cover and air temperature.

By demonstrating the contribution of the warming effect, our approach can provide an easy-to-use tool, when evaluating the increasing surface flood risk in cold climate cities in the Northern Hemisphere. The potential increases in urban flooding could have significant effects on the approximately 338 million people (based on 2010 data^31, 32^) and economic losses in these cities. To calculate realistic surface runoff for specific areas in these regions, however, it is necessary to have climatological data as well as surface cover characteristics such as irrigation and human activity profiles, which remain challenging to obtain at appropriate scales ranging from the neighbourhood to catchment^33, 34^.

## Methods

### Study areas

The 11 modelled areas had observed surface water and/or energy balance data available allowing evaluation of SUEWS output. The areas vary in surface cover with different site-specific human activities such as irrigation and building heating (See Supplementary Table S1 for site-specific parameter values). Five areas in Helsinki, with low- to high-density, were modelled (July 2005–December 2012). Conditional sampling of the eddy covariance (EC) tower (SMEAR III^35^ – Kumpula tower) data by wind direction provides Ku1-Ku3, with different degrees of urbanization (based on 1 km radius). Pi and Pa are water catchments where surface runoff has been monitored^36^. In Minneapolis-Saint Paul, conditional sampling of the EC tower (within 1 km radius circle, July 2006–April 2009) provides data for a typical North American suburb (SP1) and a golf course (SP2)^37^. In Montreal, EC masts (1 km radius) in urban (Rl) and suburban (Pr) areas were simulated (December 2007–September 2009)^38^. In Basel, two highly dense EC sites (1 km radius) were simulated (BSPR and BSPA, September 2001 to August 2002)^39^.

The warmest city, Basel, has relatively little winter precipitation (December–February) but frequent precipitation events during the spring months (March–May) (Fig. 1). Winters are consistently cold in both Minneapolis and Montreal, whereas in Helsinki variability between the years is very high. Particularly, 2007 was relatively warm in Helsinki. During spring, there is less variability among the four cities and years. The climate setting also controls the amount and length of the snow-covered period, with the coldest cities experiencing winters with the heaviest snow and the longest melting periods. In contrast, Basel only had a few hours with snow present during the period analysed.

### Observations

At nine sites, instrumentation on flux towers measured sensible and latent heat fluxes and net all-wave radiation continuously, and at two sites (Pa and Pi) flow meters monitored surface runoff at catchment storm flow discharge pipes for 28 months (2010–2012)^36^. The hourly EC fluxes are determined using commonly accepted procedures^37–40^. The intermittent runoff data were made continuous by simulating the whole periods with spatially distributed process based hydrological models with 4-min intervals and verifying the results against successfully measured events^41^. EC data from Helsinki in August 2010–December 2011 and from the less urbanized site in Montreal in January 2008–September, and runoff measurements from the more densely built Pa site in September 2010–April 2011 have been used to develop the model parameterisations.

### SUEWS model

The hydrological simulations are made using the Surface Urban Energy and Water balance Scheme (SUEWS^28^) version V2016a^29^. SUEWS is a single layer urban land surface model that simulates the surface energy and water balances at the local (neighbourhood or watershed) scale (from a few hundreds of meters to a few kilometres). The snow model in SUEWS includes the common snow related processes including snow aging via changes in snow density and albedo, snow heat storage, snow melt (also caused by liquid precipitation) and surface fraction of snow^30^.

SUEWS is forced with meteorological measurements depending on the amount of detailed forcing observations and EC flux data available for model evaluation (Supplementary Table S1). Site-specific forcing data are used for all sites in Helsinki and Minneapolis. Precipitation and air pressure in Montreal, and precipitation in Basel do not vary between sites within each respective city. All other meteorological variables are site-specific *in situ* measurements. The input precipitation includes both the liquid water and snow, and the separation between these is made in SUEWS based on the modelled surface temperature^30^. SUEWS forcing data uses hourly resolution, with model calculations made with a 5-min time step. A one month (minimum) model spin-up is used at all sites.

SUEWS runs are made using each site’s actual surface characteristics with detailed surface cover fractions, population densities, and irrigation and human activity profiles. To examine the sensitivity of the hydrological terms to the degree of urbanization, different scenarios are run for each city, using observed meteorological forcing data from one of the sites in each city (Ku1, SP1, BSPR and Rl). Eight different scenarios are run by increasing impervious surface cover (treated throughout as half paved and half buildings) from 10% to 90% (in 10% increments) with the remaining area being pervious (split in thirds between evergreen trees, deciduous trees and grass). Population densities within each simulation are linearly interpolated with the ratio of the building surface cover in the scenario to the actual fraction of the neighbourhood (note: change in population density will impact the anthropogenic heat flux density). The model parameters and performance, as evaluated against measurements, are provided in Supplementary Tables S1, S4 and S5.

### Data analysis

The analyses are undertaken by defining seasons: December–February (winter), March–May (snow melt/spring), and May–November (snow-free); and thermal regimes: cold snow (hours with snow on the ground when *S*_*WE*_ ≥ 0.01 mm and *T*_*air*_ < 0 °C), warm snow (hours when *S*_*WE*_ ≥ 0.01 mm and *T*_*air*_ > 0 °C), snow-free (hours when *S*_*WE*_ < 0.01 mm) and thermal melting period (mean daily air temperatures between 0–5 °C). In order to compare sites, we use normalised runoff and evapotranspiration where the cumulative *R* and *E* are divided by the sum of cumulative precipitation, cumulative irrigation and in the case of monthly values, initial snow state. There are several data gaps in EC observed evapotranspiration and therefore in model comparisons, only those hours when data are available are used in the cumulative *R* and *E*.

## Electronic supplementary material

Supplementary material
