# Supplementary material for: Warming effects on the urban hydrology in cold climate regions
Source: Sci Rep. 2017 Jul 19;7:5833. doi: 10.1038/s41598-017-05733-y (PMC5517421; doi:10.1038/s41598-017-05733-y)
Supplement: Supplementary file 1 — Supplementary material [file 41598_2017_5733_MOESM1_ESM.pdf]

Supporting information

Supplementary methods

Model evaluation

SUEWS has shown good performance against observed net all-wave radiation and eddy covariance (EC) measured turbulent sensible and latent heat flux densities in neighbourhoods in Vancouver (Canada), Montreal (Canada), Los Angeles (USA), Phoenix (USA), London (UK), Swindon (UK), Dublin (Ireland), Helsinki (Finland), Hamburg (Germany), Melbourne (Australia) and Singapore, and soil moisture in Vancouver, Los Angeles, London and Swindon[1-6]. The ability to include snow and high-latitude vegetation phenology in the model have been successfully evaluated in Helsinki and Montreal[4].

Here SUEWS performance is assessed with common statistical metrics: root mean square error (RMSE), normalised RMSE (nRMSE), mean bias error (MBE), Pearson correlation coefficient (r) and coefficient of determination (R^2) calculated using in MatLab. Consistent with past studies, SUEWS simulates the surface energy balance components at the studied areas from where hourly net all-wave radiation (Q^*) and EC measured turbulent fluxes of sensible (Q_H) and latent heat (Q_E) are available well (Supplementary Table S5). Model and observational uncertainties related to Q^* are generally lower than for Q_H and Q_E throughout the sites and period of analysis (snow-free, cold snow and warm snow), whereas the ranking of the turbulent fluxes varies depending on the period. When there is snow on ground, Q_E is better simulated than Q_H and in snow-free conditions vice-versa. Commonly, urban land surface models have been found to underestimate Q_E[7]. Differences in the model performance between the different sites are small. In Basel, the model performance during cold snow period is poor, but this is exacerbated by the small amount of data (N = 55). The poorest model performance is for the SP2 (golf course site), which is unsurprising given the prior development focus and evaluations of SUEWS on more built-up areas.

The development and melt of the snowpack at all sites is also well captured (Supplementary Fig. S3). The model tends to predict later snow melt than the observations. This may relate to single-point observations (in an open field) not necessarily being representative of the whole area that is simulated. Melt, especially in the shadows of trees and buildings and in snow piles, can be delayed when compared to the open field. In Basel with snow depth monitored outside the city, the observed snow period is expectedly longer than modelled, due to city-scale urban effects (e.g. urban heat island).

Global products

We estimated the snow extent for the Northern Hemisphere (for December-February 2008-2010) from the mean MODIS global monthly mean snow fraction product MOD10CM[8] with spatial resolution 0.05°. Grid points with over 80% of snow cover were included. Missing data due to continuous darkness at high-latitudes in winter were treated as snow covered grids. Urban land use (for the same 0.05°) from the Land Cover Type Climate Modeling Grid product (MCD12C1[9]) combined with the Gridded Population of the World (Version 4[10] 2010; 30 arc seconds aggregated to 0.05°) allowed the number of people living in the snow covered cities to be obtained.

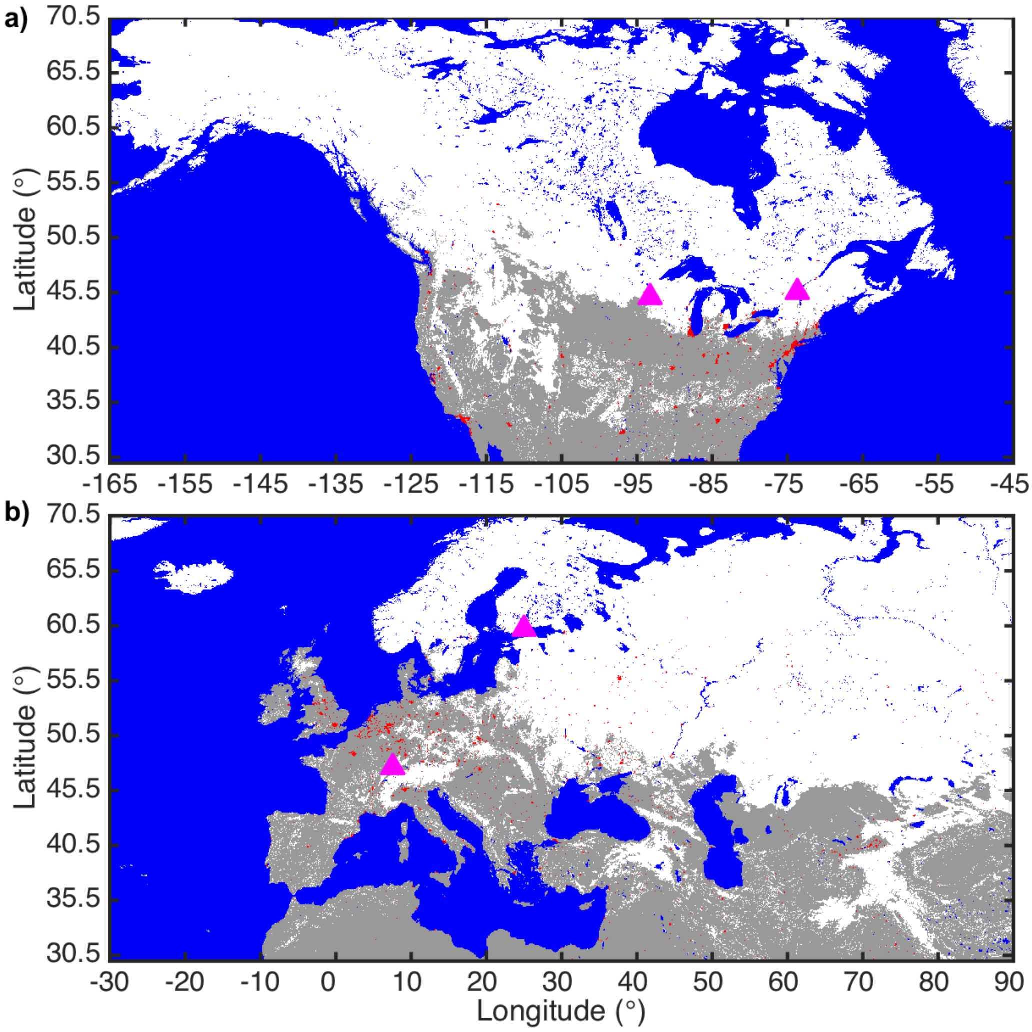

Supplementary Figure S1. Maps of snow extent and urbanisation. Snow extent (white) in December-February 2008-2010 and urbanised areas (red) in 2010 in **(a)** North-America and **(b)** Europe. Studied cities are shown with pink triangles, non-urbanised without snow in grey and water in blue. Missing data due to continuous darkness at high-latitudes in winter were treated as snow covered grids. Snow extent was defined as grids with over 80% of snow cover from mean MODIS global monthly mean snow fraction product MOD10CM[5] with spatial resolution 0.05°. Land use for the same 0.05° resolution were obtained from the Land Cover Type Climate Modeling Grid product (MCD12C1[43]). Maps were generated using MatLab V2014b (https://se.mathworks.com/products/new_products/release2014b.html).

Supplementary Table S1. Sites and parameters used in the model runs. Site-specific parameters used and initial conditions in the SUEWS model runs. See notation in Supplementary Table S6 for details.

	Helsinki					*Montreal*		*Minneapolis*		*Basel*	
Site	Ku1	Ku2	Ku3	Pa	Pi	Rl	Pr	SP1	SP2	BSPA	BSPR
Lat	60.20°N	60.20°N	60.20°N	60.20°N	60.24°N	45.46°N	45.50°N	45.00°N	45.00°N	47.55°N	47.55°N
Lon	24.96°E	24.96°E	24.96°E	24.94°E	25.01°E	73.592°W	73.81°W	93.19°W	93.19°W	7.57°E	7.57°E
Timezone	2	2	2	2	2	-5	-5	-6	-6	1	1
LCZ[11]	3	6	9	2	9	3	6	9	C	2	2
λ_{pav}	0.42	0.39	0.30	0.42	0.22	0.44	0.37	0.08	0.04	0.32	0.30
λ_{bldg}	0.20	0.15	0.11	0.20	0.12	0.27	0.12	0.12	0.01	0.37	0.54
λ_{everg}	0.01	0	0.01	0	0.10	0	0.05	0.06	0.02	0	0
λ_{dec}	0.21	0.20	0.26	0.24	0.30	0.26	0.15	0.30	0.19	0.16	0.11
λ_{grass}	0.16	0.25	0.31	0.12	0.12	0.03	0.30	0.39	0.73	0.15	0.05
λ_{bare}	0	0	0.00	0.02	0.14	0	0	0	0	0	0
λ_{water}	0	0.01	0	0	0	0	0.01	0.04	0.02	0	0
$\lambda_{Irr,grass}$	0.94	0.77	0.68	0.81	0.17	0.77	0.83	0.42	0.81	0	0
$\lambda_{Irr,trees}$	0	0	0.68	0	0	0	0	0	0	0	0
Irr. period	152-243	152-243	152-243	152-243	152-243	140-260	140-260	140-273	140-273	-	-
Fr irr_{aut}	0	0	0	0	0	0	0	0	1	-	-
z (m)	31	31	31	31	31	25	25	40	40	29.9	31.7
z_h (m)	10.4	11.5	12.6	15.2	10.8	7.9	6.4	6	6	12.5	14.6
z_t (m)	10	8.8	8.5	8	8	13.0	13.8	12	12	8.0	8.0
p (# ha^{-1})	31	37	44	42	55	84	24	10	0	103	158
A (ha)	44.7	78.2	78.2	23.8	44.8	314.2	314.2	174.5	78.5	19.6	19.6
Alt (m)	26	26	26	28	35	62	34	301	301	278	255
Reference		Karsisto et al.[12]		Järvi et al.[4]		Bergeron and Strachan[13]		Peters et al.[14]		Christen and Vogt[15]	
Period											
Modelled	7/2005-12/2012			7/2005-12/2012		12/2007-9/2009		7/2006-4/2009		9/2001-8/2002	
Analysed	7/2006-06/2012			7/2006-6/2012		7/2008-9/2009		7/2007-4/2009		10/2001-6/2002	

Initial conditions	*Units*	*Helsinki*	*Montreal*	*Minneapolis*	*Basel*
Previous day T_{air}	°C	14	-4.9	28.0	16.03
Days since rain	d	0	2	0	0
C_i	mm	10	0	10	1.5
$C_{soil,\mathrm{bldg}}$	mm	10	50	150	150
$C_{soil,\mathrm{pav}}$	mm	45	100	100	100
$C_{soil,\mathrm{veg}}$	mm	45	150	150	150
Snow on ground	-	No	No	No	No
LAI_{evetr}	$m^2\ m^{-2}$	5.1	4.0	5.1	5.1
LAI_{dectr}	$m^2\ m^{-2}$	5.5	1.0	5.5	5.5
LAI_{grass}	$m^2\ m^{-2}$	5.9	1.6	5.9	5.9
Previous day	°C	14	-4.9	28.0	16.03
Previous day T_{air}	d	0	2	0	0
Days since rain	mm	10	0	10	1.5
C_i	mm	10	50	150	150

Supplementary Table S2. Fitted coefficients between hydrological components and urbanisation. Coefficients for linear least square fits between modelled (subscript *mod*) normalised (subscript *norm*) evaporation (*E*) and runoff (*R*) against the impervious surface cover fraction. Data are stratified by winter months (Fig. 2, Supplementary Fig. S2) and thermal snow regimes – cold snow, warm snow and snow-free. Cumulative data: "All" uses all modelled data, "Observed" uses only those hours when observed evaporation are available.

	Condition	*a*	*b*	R^2	RMSE	*N*
$E_{norm,mod}$	All data – May-November	-0.642	0.809	0.71	0.073	38
$R_{norm,mod}$	All data – May-November	0.681	0.144	0.74	0.072	38
$E_{norm,obs}$	Observed data only – May-November	-0.083	0.151	0.07	0.061	23
$E_{norm,obs}$	Observed data only – May-November	-2.141	0.216	0.32	0.064	23
$R_{norm,obs}$	Observed data only – May-November	0.717	-0.065	0.53	0.133	4
$R_{norm,mod}$	Observed data only – May-November	0.414	0.181	0.20	0.196	4
$E_{norm,mod}$	Observed data only – cold snow	-0.019	0.027	0.04	0.018	20
$E_{norm,obs}$	Observed data only – cold snow	0.021	0.012	0.04	0.020	20
$E_{norm,mod}$	Observed data only – warm snow	0.040	0.014	0.08	0.026	17
$E_{norm,obs}$	Observed data only – warm snow	0.060	0.003	0.16	0.027	17
$E_{norm,mod}$	Observed data only – snow free	-0.253	0.205	0.35	0.0727	21
$E_{norm,obs}$	Observed data only – snow free	-0.098	0.123	0.12	0.0575	21

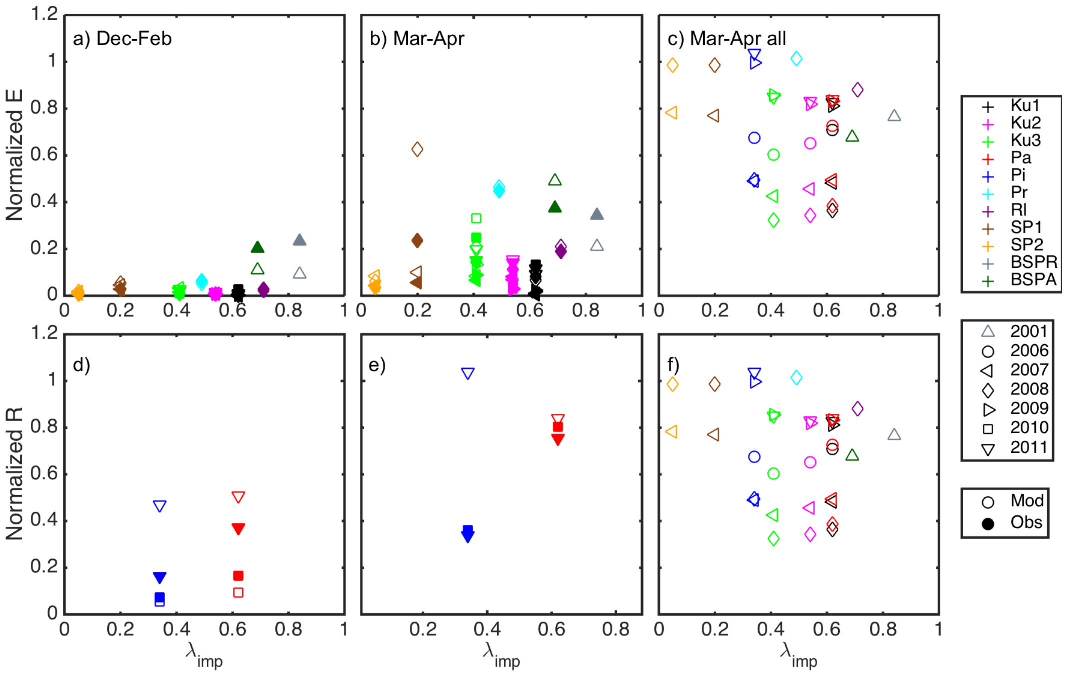

Supplementary Figure S2. Hydrological components with impervious cover. Cumulative **(a-c)** evapotranspiration (*E*) and **(d-f)** surface runoff (*R*) as normalised by cumulative precipitation and irrigation. **(a, d)** show modelled (open symbols) and measured (closed symbols) winter months (December-February) and **(b, e)** spring months (March-April) so that in cumulative *E* and *R* only hours with observed data are used, and **(c, f)** modelled spring months for all hours. Different sites (colours) and years (symbols) are indicated for the observations (solid symbols) and modelled (open) values.

Supplementary Table S3. Fitted coefficients between hydrological components and climate and urbanization. Coefficients for non-linear least square fit to runoff and evaporation as a function of air temperature and fraction of snow days for winter (data analyzed months or 2-week periods). The fitted curve has an exponential form ($aexp(bT_{air})$) for the normalised evapotranspiration, and a logistic form ($\frac{a}{1+\exp[-b(T_{air}-c)]}$) for the normalised runoff

λ_{imp}	*a*	*b*	*c*	R^2	RMSE	*N*
Normalized monthly R versus T_{air}						
10%	0.562±0.093	1.064±1.107	-3.012±1.205	0.78	0.124	37
20-30%	0.598±0.060	0.996±0.582	-2.964±0.742	0.82	0.112	74
40-50%	0.654±0.057	0.909±0.408	-2.886±0.649	0.87	0.099	74
60-70%	0.718±0.555	0.810±0.278	-2.766±0.586	0.91	0.089	74
80-90%	0.801±0.062	0.675±0.181	-2.584±0.593	0.93	0.085	74
Normalized 2-week R versus T_{air}						
10%	0.552±0.119	1.106±1.354	-2.532±1.263	0.44	0.260	83
20-30%	0.590±0.077	0.954±0.618	-2.529±0.811	0.52	0.228	166
40-50%	0.657±0.070	0.780±0.346	-2.450±0.725	0.64	0.192	166
60-70%	0.732±0.070	0.615±0.197	-2.330±0.713	0.72	0.171	166
80-90%	0.838±0.080	0.472±0.121	-2.043±0.790	0.75	0.170	166
Normalized December-February R versus T_{air}						
10%	0.523±0.169	0.773±0.934	-5.201±3.415	0.88	0.095	10
20-30%	0.558±0.094	0.705±0.398	-5.177±1.665	0.90	0.081	20
40-50%	0.616±0.091	0.619±0.265	-0.056±1.358	0.92	0.074	20
60-70%	0.685±0.092	0.535±0.180	-4.847±1.168	0.94	0.069	20
80-90%	0.780±0.105	0.453±0.131	-4.464±1.126	0.95	0.067	20
Monthly normalized E versus T_{air}						
10%	0.259±0.061	0.125±0.046	-	0.49	0.142	37
20-30%	0.239±0.037	0.118±0.030	-	0.48	0.124	74
40-50%	0.209±0.030	0.107±0.028	-	0.46	0.102	74
60-70%	0.177±0.023	0.090±0.025	-	0.42	0.080	74
80-90%	0.140±0.017	0.063±0.022	-	0.31	0.057	74
Occurrence on intense daily runoff events as a function of T_{air} in December-February						
10-90%	3.479±0.814	2.509±40.88	-3.676±18.114	0.49	1.758	90
Occurrence on intense daily runoff events as a function of S_{WE} in thermal spring						
10-90%	0.0592±0.001	-0.185±0.441	-	0.61	1.571	108

Supplementary Table S4. Model parameters used in the SUEWS model runs. See notation in Supplementary Table S6 and SUEWS manual (http://urbanclimate.net/umep/SUEWS) for details.

	Site	Units	Bldg	Pav	Everg	Decid	Grass	Bare soil	Water
S_i	All	mm	0.25	0.48	1.3	0.3-0.8	1.9	1.0	2000
$S_{soil,i}$	Hel, Basel, RI	mm	50	100	150	150	150	150	-
	Pr, SP1, SP2	mm	150	100	150	150	150	150	-
$D_{0,i}$	All	mm	10	10	0.013	0.013	0.013	0.013	-
d	All	-	3	3	1.71	1.71	1.71	1.71	-
α_i	All	-	0.15	0.09	0.10	0.16	0.19	0.21	0.08
ε_i	All	-	0.95	0.91	0.98	0.98	0.93	0.94	0.95
LAI	All	$m^2\ m^{-2}$	-	-	4.0-5.1	1.0-5.5	1.6-5.9	-	-
$g_{i,\max}$	All	$mm\ s^{-1}$	-	-	7.4	11.7	40	-	-
$S_{WE,i}^{\max}$	All	mm	190	190	190	190	190	190	-
$S_{WE,\lim}$	All	mm	40	100	-	-	-	-	-
a_1	Helsinki		0.19	0.719	0.11	0.11	0.32	0.355	0.5
	RI		0.26	0.719	0.11	0.11	0.32	0.355	0.5
	Pr		0.12	0.719	0.11	0.11	0.32	0.355	0.5
	Minneapolis		0.238	0.719	0.11	0.11	0.32	0.355	0.5
	Basel		0.238	0.719	0.336	0.336	0.32	0.355	0.5
a_2	Helsinki		0.54	0.194	0.11	0.11	0.54	0.355	0.21
	RI		0.85	0.194	0.11	0.11	0.54	0.355	0.21
	Pr		0.24	0.194	0.11	0.11	0.54	0.355	0.21
	Minneapolis		0.427	0.194	0.11	0.11	0.54	0.355	0.21
	Basel		0.427	0.194	0.313	0.313	0.54	0.355	0.21
a_3	Helsinki		-15.125	-36.6	-12.3	-12.3	-27.4	-35.275	-39.1
	RI		-21.4	-36.6	-12.3	-12.3	-27.4	-35.275	-39.1
	Pr		-4.5	-36.6	-12.3	-12.3	-27.4	-35.275	-39.1
	Minneapolis		-16.7	-36.6	-12.3	-12.3	-27.4	-35.275	-39.1
	Basel		-16.7	-36.6	-31.4	-31.4	-27.4	-35.275	-39.1

b) Overall area parameter values

Snow		*Surface conductance (others/Basel)*		*Vegetation phenology*		*Anthropogenic heat*	
$\alpha_s^{\min}$	0.18	G_1	16.48/3.5 mm s^{-1}	GDD	300	$a_{0,wd/we}$	0.1 W m^{-2} (p^{-1} ha^{-1})$^{-1}$
$\alpha_s^{\max}$	0.85	G_2	566.1/200 W m^{-2}	b_1 Bas	0.03	$a_{1,wd/we}$	9.9·10^3 $Wm^{-2}K^{-1}$ (p^{-1} ha^{-1})$^{-1}$
ε_s	0.99	G_3	0.216/0.13 kg g^{-1}	b_1 others	0.04	$a_{2,wd/we}$	0.0102 $Wm^{-2}K^{-1}$ (p^{-1} ha^{-1})$^{-1}$
ρ_e	200 kg m^{-3}	G_4	3.36/0.7 g kg^{-1}	c_1 Bas	0.0005	T_{BaseQF}	18.2°C
$\rho_s^{\min}$	100 kg m^{-3}	G_5	11.07/30°C	c_1 others	0.001	*Others*	
$\rho_s^{\max}$	400 kg m^{-3}	G_6	0.018/0.05 mm	b_2 Basel	0.03	K_s	0.0005 mm s^{-1}
τ_a	0.018	$K\downarrow_m$	1200 W m^{-2}	b_3 Others	-1.5	$R_{s,max}$	9999 s m^{-1}
τ_f	0.11	S_1	0.45/5.56 mm	c_2 Basel	0.0005	res_{cap}	10 mm
τ_r	0.043	S_2	15/0 mm	c_3 Others	0.0015	res_{drain}	0.25 mm h^{-1}
a_1	0.25	T_H	40/50°C	SDD	-450	S_{Pipe}	100 mm
a_2	0.6	T_L	0/-10°C	$T_{BaseGDD}$	5°C	T_{step}	300 s
a_3	-30	*Irrigation*		$T_{BaseSDD}$	10°C		
a_f	1	$b_{0,a}$	-19.1853 mm				
a_r	0.0016 mm W^{-1} h^{-1}	$b_{1,a}$	2.2195 mm K^{-1}				
a_t	0.07 mm °C^{-1} h^{-1}	$b_{2,a}$	0.7836 mm d^{-1}				
$C_{\min}^R$	0.05 mm	$b_{0,m}$	-5.7556 mm				
$C_{\max}^R$	0.2 mm	$b_{1,m}$	0.6658 mm K^{-1}				
P_{lim}	2 mm	$b_{2,m}$	0.2351 mm d^{-1}				
T_{lm}	2.2°C	I_w	0 mm				

Supplementary Table S5. Model evaluation metrics. Summary of the metrics at all sites (excluding Pa and Pi) with hourly net all-wave radiation (Q^*), sensible (Q_H) and latent heat flux densities (Q_E) by thermal snow regimes. RMSE = root mean square error (W m^{-2}), nRMSE = normalized root mean square error with the difference between observed maximum and minimum, MBE = mean biased error (W m^{-2}), r = Pearson correlation coefficient, and *N* = number of points (only periods with more than five points are calculated).

		Cold snow			Warm snow			Snow-free		
		Q^*	Q_H	Q_E	Q^*	Q_H	Q_E	Q^*	Q_H	Q_E
He1	RMSE	29.7	28.2	4.7	39.4	43.2	16.5	34.2	39.1	23.1
	nRMSE	0.051	0.066	0.041	0.047	0.090	0.141	0.027	0.059	0.057
	MBE	-18.9	-23.2	-4.7	-4.8	-9.9	-7.2	7.3	-23.7	-20.1
	r	0.74	0.74	0.59	0.94	0.83	0.65	0.98	0.89	0.72
	N	12495	1257	984	6659	393	346	42201	2398	1926
He2	RMSE	29.3	30.6	4.1	38.8	39.5	18.4	34.0	52.7	32.4
	nRMSE	0.051	0.077	0.028	0.046	0.083	0.112	0.027	0.078	0.079
	MBE	-19.0	-7.4	-5.5	-5.3	9.3	2.9	6.8	-8.8	-18.9
	r	0.74	0.61	0.48	0.94	0.77	0.70	0.98	0.85	0.63
	N	12499	2424	942	6667	801	409	42189	5414	3639
He3	RMSE	29.5	28.7	5.7	38.1	44.2	35.2	33.7	51.5	50.4
	nRMSE	0.051	0.066	0.065	0.046	0.079	0.203	0.027	0.083	0.111
	MBE	-18.9	6.0	-2.3	-6.2	14.3	12.1	5.7	-0.4	14.7
	r	0.74	0.56	0.49	0.94	0.75	0.65	0.98	0.74	0.81
	N	12519	1518	532	6774	1700	810	42062	11918	7071
PR	RMSE	36.8	30.7	10.4	70.2	60.6	43.6	80.0	52.6	41.2
	nRMSE	0.053	0.078	0.035	0.091	0.133	0.184	0.090	0.114	0.079
	MBE	-44.0	-4.3	-1.6	-13.3	8.0	11.0	3.0	3.6	-11.8
	r	0.89	0.86	0.52	0.93	0.81	0.57	0.93	0.85	0.74
	N	2990	2178	2063	1591	998	972	6494	5269	5138
RL	RMSE	42.3	32.0	7.2	71.8	48.4	28.3	85.2	51.7	30.3
	nRMSE	0.063	0.070	0.054	0.096	0.100	0.128	0.103	0.093	0.070
	MBE	-27.1	11.5	-2.9	-6.3	12.6	3.3	17.5	16.9	-14.1
	r	0.86	0.87	0.59	0.91	0.89	0.36	0.93	0.91	0.50
	N	2713	1371	1289	1375	788	759	4138	3491	3404
SP1	RMSE	47.8	28.2	5.7	56.2	61.0	53.1	36.9	75.9	72.6
	nRMSE	0.070	0.065	0.039	0.081	0.100	0.239	0.046	0.096	0.113
	MBE	-4.0	-1.3	3.0	3.6	-4.7	27.0	18.9	2.9	16.7
	r	0.74	0.82	0.37	0.95	0.73	0.71	0.98	0.79	0.58
	N	6576	3564	3564	2117	824	824	10364	4064	4064
SP2	RMSE	47.4	29.1	3.7	55.6	92.2	112.5	36.1	118.4	122.2
	nRMSE	0.070	0.090	0.038	0.080	0.158	0.155	0.045	0.217	0.187
	MBE	-4.4	3.5	2.4	-0.1	8.5	17.1	16.7	-4.2	33.8
	r	0.75	0.77	0.48	0.95	0.27	0.12	0.98	0.34	0.63
	N	6611	1231	1231	2150	364	364	10296	1759	1759
BSPR	RMSE	16.2	51.5	1.3	-	-	-	28.7	42.1	15.5
	nRMSE	0.057	0.272	0.087	-	-	-	0.035	0.066	0.023
	MBE	50.0	80.7	-22.4	-	-	-	4.0	6.2	-14.0
	r	0.99	0.52	0.60	-	-	-	0.98	0.91	0.64
	N	27	7	6	-	-	-	5659	3806	2566
BSPA	RMSE	24.7	44.8	0.8	-	-	-	27.5	38.0	32.1
	nRMSE	0.074	0.368	0.006	-	-	-	0.034	0.069	0.035
	MBE	39.2	32.1	-23.8	-	-	-	9.7	5.4	2.5
	r	0.97	0.72	-0.11	-	-	-	0.98	0.87	0.45
	N	49	45	27	-	-	-	7962	6492	4225

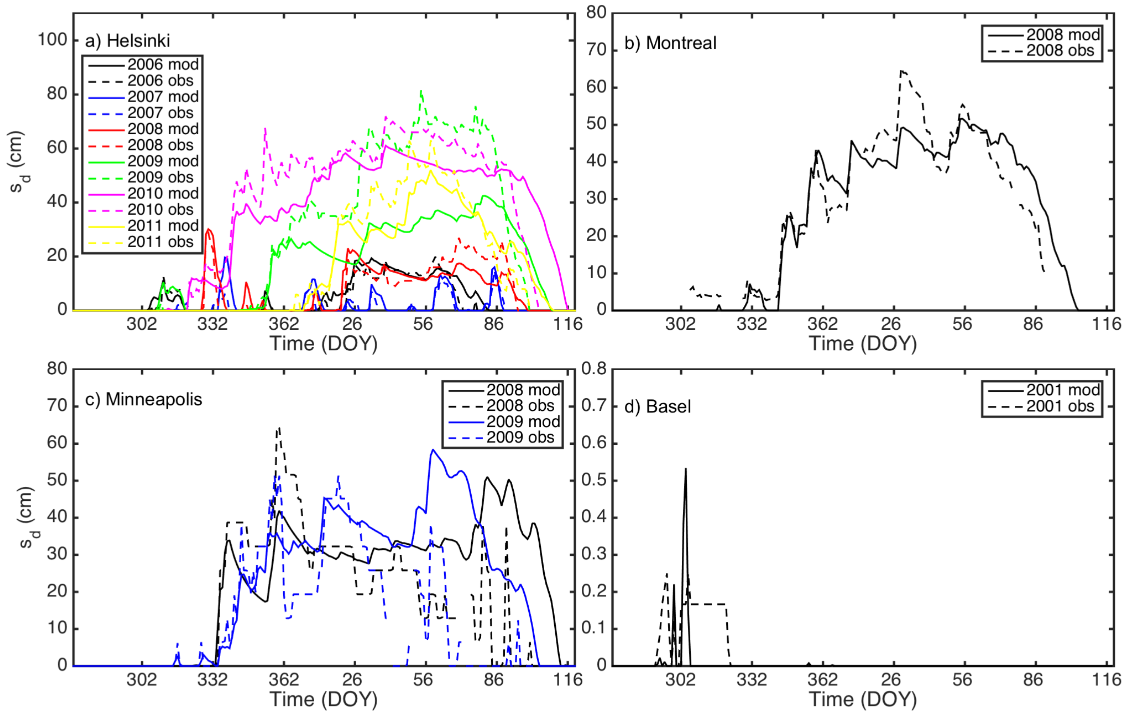

Supplementary Figure S3. Modelled and measured snow depths (s_d). **(a)** In Helsinki (He3), **(b)** Montreal (RI), **(c)** Minneapolis (SP1) and **(d)** Basel (BSPA). In Basel, where snow depth was only monitored outside the city, the observed snow period is expectedly longer than modelled, due to regional urban effects (e.g. urban heat island). Modelled values are for the grass surface as these are most representative of the snow observations made in open spaces (according to the WMO[16,17] recommendations).

Supplementary Table S6. Notation in alphabetical order

Abbreviation	Description
α_i	Effective surface albedo (-)
$\alpha_s^{\min}$	Minimum snow albedo (-)
$\alpha_s^{\max}$	Maximum snow albedo (-)
ε_i	Effective surface emissivity (-)
ε_s	Effective surface emissivity of snow (-)
λ_{bsoil}	Surface fraction of bare soil (-)
λ_{bldg}	Surface fraction of buildings (-)
λ_{dectr}	Surface fraction of deciduous trees (-)
λ_{evetr}	Surface fraction of evergreens (-)
λ_{grass}	Surface fraction of non-irrigated grass (-)
λ_{imp}	Surface fraction of impervious surfaces (-)
λ_{pav}	Surface fraction of paved areas (-)
λ_{veg}	Surface fraction of vegetation (-)
λ_{water}	Surface fraction of water (-)
ρ_e	Threshold in the calculation of snow retention capacity (kg m^{-3})
$\rho_s^{\min}$	Maximum snow density (kg m^{-3})
$\rho_s^{\max}$	Minimum snow density (kg m^{-3})
τ_a	Cold snow time constant for snow albedo aging (-)
τ_f	Warm snow time constant for snow albedo aging (-)
τ_r	Time constant describing the snow density aging (-)
a	Fitting coefficient
$a_{0,wd/we}$	Parameter defining the base anthropogenic heat flux (W m^{-2} (cap^{-1} $ha^{-1})^{-1}$)
$a_{1,wd/we}$	Parameter related to CDD (W m^{-2} K^{-1} ($capita^{-1}$ $ha^{-1})^{-1}$)
$a_{2,wd/we}$	Parameter related to HDD (W m^{-2} K^{-1} ($capita^{-1}$ $ha^{-1})^{-1}$)
$a_{1,2,3}$	Constants in the calculation of the heat storage in OHM
a_f	Temperature freezing factor (mm $°C^{-1}$ h^{-1})
a_r	Radiation melt factor (mm W^{-1} h^{-1})
a_t	Temperature melt factor (mm $°C^{-1}$ h^{-1})
A	Study area (ha)
Alt	Altitude (m)
b	Fitted coefficient
d	Empirical coefficient in the calculation of drainage
$b_{1,2,3}$	Parameters controlling the speed of leaf on period
$b_{1a,2a,3a}$	Parameters for automatic irrigation (mm, mm K^{-1}, mm d^{-1})
$b_{1m,2m,3m}$	Parameters for manual irrigation (mm, mm K^{-1}, mm d^{-1})
bldg	Building surface type
BSPA	Spalenring site in Basel, Switzerland
BSPR	Sperrstrasse site in Basel, Switzerland
c	Fitted coefficient
$c_{1,2,3}$	Parameter to control the speed of leaf-off
C_i	Initial surface state of *i*th surface type (mm)
$C_{soil,i}$	Initial soil water state of *i*th surface type (mm)
$C_{\min}^R$	Minimum retention capacity of snow (mm)
$C_{\max}^R$	Maximum retention capacity of snow (mm)
d	Empirical coefficient in the calculation of drainage
$D_{0,i}$	Drainage rate of *i*th surface type (mm)
dectr	Deciduous surface type
E	Evaporation (mm)
E_{norm}	Normalised evaporation
EC	Eddy covariance

evetr	Evergreen surface type
$g_{i,max}$	Maximum conductance (m s^{-1})
G_{1-6}	Parameters related to surface conductance
GDD	Growing degree days
i	Surface type index (bldg, pav, evetr, dectr, grass, bsoil, water)
I_e	Irrigation
I_w	Amount of water for internal water use
$K{\downarrow}_{\mathrm{m}}$	Maximum incoming solar radiation used in g_s calculation
K_s	Saturated hydraulic conductivity of soil (mm s^{-1})
Ku1	Built sector at the Kumpula site
Ku2	Road sector at the Kumpula site
Ku3	Vegetation sector at the Kumpula site
LAI	Leaf area index (m^2 m^{-2})
Lat	Latitude (°)
LCZ	Local climate zone
Lon	Longitude (°)
MBE	Mean biased error
nRMSE	Normalised root mean square error
N	Number of data points
OHM	Objective hysteresis model to calculate storage heat flux
p	Population density inside the grid (capita ha^{-1})
P	Precipitation (mm)
P_{lim}	Snowfall limit when the surface albedo is changed to fresh snow albedo (mm)
Pa	Pasila site in Helsinki, Finland
pav	Paved surface type
Pi	Pihlajamäki site in Helsinki, Finland
Pr	Pierrefonds-Roxboro site in Montreal, Canada
Q^*	Net all-wave radiation (W m^{-2})
Q_E	Latent heat flux density (W m^{-2})
Q_H	Sensible heat flux density (W m^{-2})
r	Pearson correlation coefficient
$r_{s,max}$	Maximum surface resistance (s m^{-1})
res_{cap}	Surface water capacity in LUMPS (mm)
res_{drain}	Drainage rate of water bucket in LUMPS (mm h^{-1})
R	Runoff (mm)
R_{norm}	Normalised runoff
R^2	Squared root mean square error
R_C	Limit when surface is totally covered with water in LUMPS (mm)
Rl	Rosemont-La-Petite-Patrie site
RMSE	Root mean square error
s_d	Snow depth (m)
S_{1-2}	Parameters related to surface conductance
S_{days}	Number of snow days
S_i	Water state of the snow free surface (mm)
S_{Pipe}	Maximum depth capacity of pipes (mm)
$S_{soil,i}$	Soil state (mm)
S_{WE}	Snow water equivalent (mm)
$S_{WE,Lim}$	S_{WE} limit for snow removal from the area (mm)
$S_{WE,i}^{\max}$	S_{WE} when surface type i is fully covered with snow (mm)
SDD	Senescence degree days
SP1	Suburban surface area in Minneapolis-St. Paul in USA
SP2	Simulated golf course in Minneapolis-St. Paul in USA.
SUEWS	The Surface Urban Energy and Water balance Scheme
T_{air}	Air temperature (°C)
$T_{BaseGDD}$	Base temperature for leaf growth (°C)
$T_{BaseSDD}$	Base temperature for senescence (°C)
T_{BaseQF}	Base temperature for Q_F (°C)
T_H,T_L	Maximum and minimum temperature limits in calculation of g_s (°C)

T_{lim}	Temperature limit for the liquid precipitation and snow (°C)
T_{Step}	Model time step (s)
z	Height of the meteorological measurements (m)
z_b	Mean building height (m)
z_t	Mean tree height (m)

References

1 Ward, H. C., Kotthaus, S., Järvi, L. & Grimmond, C. S. B. Surface urban energy and water balance scheme (SUEWS): development and evaluation at two UK sites. *Urban Climate* **18**, 1-32 (2016).

2 Alexander, P. J., Fealy, R. & Mills, G. M. Simulating the impact of urban development pathways on the local climate: A scenario-based analysis in the greater Dublin region, Ireland. *Landscape Urban Plan* **152**, 72-89 (2016).

3 Järvi, L., Grimmond, C. S. B. & Christen, A. The Surface Urban Energy and Water Balance Scheme (SUEWS): Evaluation in Los Angeles and Vancouver. *Journal of Hydrology* **411**, 219 - 237, doi:10.1016/j.jhydrol.2011.10.001 (2011).

4 Järvi, L. *et al.* Development of the Surface Urban Energy and Water Balance Scheme (SUEWS) for cold climate cities. *Geoscientific Model Development* **7**, 1691 - 1711, doi:10.5194/gmd-7-1691-2014 (2014).

5 Alexander, P. J., Bechtel, B., Chow, W. T. L., Fealy, R. & Mills, G. Linking urban climate classification with an urban energy and water budget model: Multi-site and multi-seasonal evaluation. *Urban Climate* **17**, 196-215, doi:10.1016/j.uclim.2016.08.003 (2016).

6 Demuzere, M. *et al.* Impact of urban canopy models and external parameters on the modelled urban energy balance in a tropical city. *Quarterly Journal of the Royal Meteorological Society*, doi:10.1002/qj.3028 (2017).

7 Grimmond, C. S. B. *et al.* Initial results from Phase 2 of the international urban energy balance model comparison. *International Journal of Climatology* **31**, 244 - 272, doi:DOI: 10.1002/joc.2227 (2011).

8 Hall, D. K. & A, R. G. MODIS/Terra Snow Cover Monthly L3 Global 0.05Deg CMG, Version 6. [MOD10CM]. (NASA National Snow and Ice Data Center Distributed Active Archive Center, 2015).

9 NASA LP DAAC. MCD12C1 The Land Cover Type Climate Modeling Grid product., (NASA EOSDIS Land Processes DAAC, USGS Earth Resources Observation and Science (EROS) Center, Sioux Falls, South Dakota (https://lpdaac.usgs.gov), 2015).

10 Center for International Earth Science Information Network - CIESIN - Columbia University. Documentation for the Gridded Population of the World, Version 4 (GPWv4). (Palisades NY: NASA Socioeconomic Data and Applications Center (SEDAC), 2016).

11 Stewart, I. D. & Oke, T. R. Local Climate Zones for Urban Temperature Studies. *Bulletin of the American Meteorological Society* **93**, 1879 - 1900, doi:DOI: 10.1175/bams-d-11-00019.1 (2012).

12 Karsisto, P. *et al.* Seasonal surface urban energy balance and wintertime stability simulated using three land-surface models in the high-latitude city Helsinki. *Quarterly Journal of the Royal Meteorological Society* **142**, 401-417, doi:10.1002/qj.2659 (2016).

13 Bergeron, O. & Strachan, I. B. Wintertime radiation and energy budget along an urbanization gradient in Montreal, Canada. *International Journal of Climatology* **32**, 137-152, doi:10.1002/joc.2246 (2012).

14 Peters, E. B., Hiller, R. V. & McFadden, J. P. Seasonal contributions of vegetation types to suburban evapotranspiration. *Journal of Geophysical Research-Biogeosciences* **116**, doi:10.1029/2010jg001463 (2011).

15 Christen, A. & Vogt, R. Energy and radiation balance of a central European city. *International Journal of Climatology* **24**, 1395-1421, doi:10.1002/joc.1074 (2004).

16 WMO. Guide to Hydrological Practises (5th edition). (Geneva, 1994).

17 WMO. Guide to Meteorological Instruments and Methods of Observation (7th Edition). (Geneva, 2008).
